# Down-regulation of FTO promotes proliferation and migration, and protects bladder cancer cells from cisplatin-induced cytotoxicity

**DOI:** 10.1186/s12894-020-00612-7

**Published:** 2020-04-16

**Authors:** Lijie Wen, Xianwei Pan, Yang Yu, Bo Yang

**Affiliations:** 1 Department of Urology, The Second Hospital of Dalian Medical University, 467 Zhongshan Road, Shahekou, Dalian, 116023 China; 2 Department of Library, Dalian Party Institute of CPC, Dalian, China

**Keywords:** FTO, Bladder urothelial carcinoma, Meclofenamic acid, Cisplatin

## Abstract

**Background:**

FTO is known to be associated with body mass and obesity in humans and its over-expression affects the energy metabolism of cancer cells. The aim of the present study is to investigate the biological role of FTO in human bladder urothelial carcinoma.

**Methods:**

PCR and western blotting are used to measure the levels of FTO in both tissues and cell lines (5637, T24, TCCSUP) of human bladder urothelial carcinoma. Raw RNA-Sequencing reads and the corresponding clinical information for bladder urothelial carcinoma are downloaded from TCGA. Cell Counting Kit-8 and wound healing assays are used to explore the effect of FTO on proliferation and migration of bladder cancer cells.

**Results:**

The expression of FTO mRNA in bladder urothelial carcinoma decreases significantly compared with the normal controls from both the data of real-time PCR (*p* < 0.05) and TCGA (*p* < 0.01). Loss-of-function assays revealed that knockdown of FTO significantly promotes proliferation and migration of 5637 and T24 cells. Consistently, we found that the cisplatin-induced cytotoxicity of bladder cancer cell could be rescued by co-treatment with MA2, which was previously reported as a highly selective inhibitor of FTO, compared with the cisplatin-control group.

**Conclusions:**

These findings suggest that down-regulation of FTO plays an oncogenic role in bladder cancer. The further exploration of regulation of FTO expression may provide us a potential therapeutic target for the treatment of bladder cancer.

## Background

Bladder cancer (BC) is one of the most common type of cancer worldwide. Urothelial (transitional) cell carcinoma is the most common type of bladder cancer. Bladder cancer exhibits a high relapse frequency and a poor clinical outcome once the tumor progresses to muscle-invasive disease, which could result in significant economic impact [1, 2]. The survival rate is still not significantly improved in spite of the diverse treatment options available for patients with bladder cancer (surgery, chemotherapy and radiation therapy). Tumor stage (TNM) is recognized as one of the strongest prognostic factors in the clinical outcome of patients with BC. In a surgery-only study, the five-year recurrence-free survival was 76% in patients with pT1 tumours, 74% for pT2, 52% for pT3, and 36% for pT4 [3]. However, in some cases, even when tumors present similar histology, they respond differently to the same treatment, resulting in the differential survival of patients [4].

There are more than 150 types of posttranscriptional modifications identified in RNA among all living organisms [5]. RNA methylation of gene expression modulation is an aspect of physiological development, and its dysregulation is involved in carcinogenesis [6]. N6-methyladenosine (m6A) is most abundant among all the modification in eukaryotic cells [7, 8]. Epigenetic modifications could affect gene expression without altering the DNA structure, which offer us additional therapeutic options. The fat mass and obesity-associated protein (FTO) is known to be associated with body mass and obesity in humans and its over-expression affects the energy metabolism of cancer cells [9, 10]. The belief that FTO could affect human obesity led to intense interest in understanding its function. Analysis of the amino acid sequence of FTO showed that it contained sequence motifs which were found in the ALKB family of dioxygenase enzymes and may function as an alpha-ketoglutarate dependent dioxygenase [11]. In the study of Jia et al., there were decreases in m6A in poly(A) mRNA in cells that overexpressed FTO, and increases in m6A in FTO-knockdown cells [12]. Recently, FTO has been proved to play a critical oncogenic role in AML as a N6-methyladenosine RNA demethylase [13]. These results propose that FTO-mediated m6A demethylation might serve as a mRNA regulatory mechanism. In the present study, we intend to investigate the biological role of fat mass and obesity associated protein (FTO) in human bladder urothelial carcinoma.

## Methods

### Tissue collection and ethical statement

Bladder urothelial carcinoma samples and the adjacent normal controls were collected from 30 patients (paired) following radical cystectomy in the Second Hospital of Dalian Medical University between September 2017 and March 2018. All histologic diagnoses were conducted by the pathology department of the Second Hospital of Dalian Medical University. Informed consents were signed by all the patients involved. This study were permitted by the Ethics Committee at the Second Hospital of Dalian Medical University (2017-EC-055).

### Cell culture and transfection

5637, T24, TCCSUP and SV-HUC-1 cells were purchased from the Cell Bank of Type Culture Collection (Chinese Academy of Sciences, Shanghai, China). 5637 and T24 cells were cultured in RPMI 1640 medium. Dulbecco’s modified eagle medium and F12K medium were used to culture TCCSUP and SV-HUC-1cells (the normal bladder epithelial cell line) respectively. All mediums contained 10% fetal bovine serum and 1% penicillin and streptomycin. Cells were cultured in a humidified atmosphere with 5% CO_2_ at 37 °C.

Small interfering RNAs (siRNAs) targeting FTO were purchased from Shanghai GenePharma Co., Ltd. (Shanghai, China) and transfected into cancer cells with Lipofectamine 3000 (Invitrogen; Thermo Fisher Scientific, Inc.) according to the manufacturer’s instructions. SiRNA sequence 1: 5′ -AAAUAGCCGCUGCUUGUGAGA-3′; siRNA sequence 2: 5′ -UCUCACAAGCAGCGGCUAUUU-3′.

### Reverse transcription-quantitative PCR (RT-qPCR) assays

Total RNA of bladder cancer cell lines and samples were extracted using TRIzol reagent according to the manufacturer’s instructions. cDNA was obtained using the PrimeScript™ RT reagent kit supplied by Takara (Dalian, China). Quantitative polymerase chain reaction (PCR) was performed in Applied Biosystems QuantStudio 3 system (Thermo Fisher Scientific, USA) using the corresponding PCR reagent from Takara (Dalian, China). Primer for FTO are 5′-AATAGCCGCTGCTTGTGAGA-3′ (forward) and 5′-CAATGGCACAGCATCCTCAT-3′ (reverse). Primer for β-actin are 5′-CCGTGAAAAGATGACCCAGATC-3′ (forward) and 5′-CACAGCCTGGATGGCTACGT-3′ (reverse). Real-time PCR was performed as follows: 95 °C for 30 s, followed by 40 cycles of 95 °C for 5 s and 60 °C for 34 s to anneal. 2^− ΔΔCq^ method was used to quantify the relative expression of FTO mRNA.

### Western blotting

Cells were lysed using lysis buffer (8 mol/L urea, 1 mmol/L dithiothreitol, 1 mmol/L ethylenediaminetetraacetic acid, 50 mmol/L Tris-HCl, pH 8.0). Lysates were incubated on ice for 30 min and then sonicated for 15 s two times. The lysate was centrifuged at 12,000 rpm for 5 min at 4 °C. Supernatant was recovered and the protein concentration was measured using Pierce Micro BCA Protein Assay Kit (Thermo Fisher Scientific, Waltham, MA). The equal amount of protein samples were separated by 10% SDS-PAGE and electrotransferred onto nitrocellulose membranes. After blocking with 3% BSA in TBST, the membranes were incubated with FTO (Cell Signaling Technology, MA, USA) and GAPDH (Cell Signaling Technology, MA, USA) primary antibody overnight at 4 °C. After washing, the membranes were probed with anti-rabbit (Abcam) secondary antibodies conjugated with horseradish peroxidase for 1 h in room temperature. Bands were measured using the Quality One analysis software and the density of each band was normalized to GAPDH.

### Cell proliferation assay

Cell proliferation assay was performed as described in our experiments previously [14]. Briefly, after transfection with control or targeting siRNA for 16 h, T24 and 5637 cells were plated in 96-well plates at 1.0 × 10^4^ and 1.5 × 10^4^ cells/well in triplicates respectively. Cell proliferation was evaluated at 0, 24, 48 and 72 h after transfection using a CCK-8 assay according to the manufacturer’s protocol. The experiment was repeated at least three times.

### Cell migration assay

Wound healing assays were performed as previously described [14].

### Drugs and reagents

Cisplatin was purchased from Hansoh Pharma, Jiangsu, China and the compound MA2 was a gift from Professor CaiGuang Yang (CAS Key Laboratory of Receptor Research, Shanghai Institute of Materia Medica, Chinese Academy of Sciences, Shanghai, China). MA2 was diluted in DMSO (Solarbio, Beijing, China) to a stock concentration of 50 mM.

### TCGA database and statistical analysis

We downloaded gene expression data by RNA-seq and the corresponding clinical data for a total of 412 cancerous (bladder urothelial carcinoma) and 19 normal samples from TCGA. All RNA expression levels of the samples were normalized. The significance of differences of FTO mRNA expression between different stages of bladder urothelial carcinoma and the normal controls was assessed using a one-way ANOVA with Tukey’s tests. The differential expression levels of FTO mRNA between the cancerous and adjacent tissues were analyzed with t-tests. The results were analyzed using GraphPad Prism 7.0 and the SPSS 23.0 software. All statistical tests were two-tailed, with *p* < 0.05 considered significant.

## Results

### FTO expression in bladder urothelial carcinoma tissues and cell lines

**Fig. 1:**
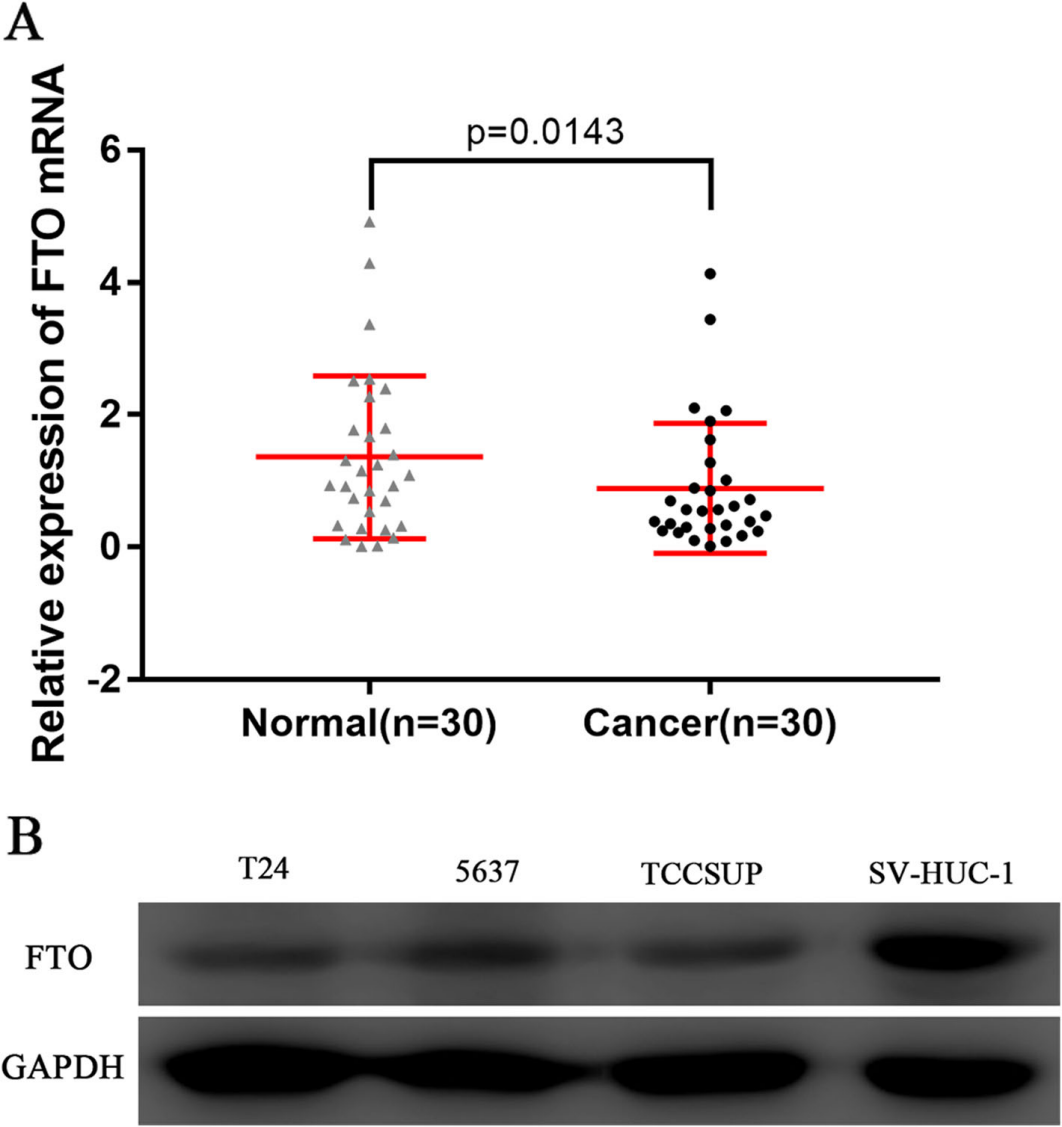
FTO expression in tissues and cell lines of bladder urothelial carcinoma**. a** Relative expression of FTO mRNA in bladder urothelial carcinoma tissues and the normal controls. The expression values are log10-transformed and mean centered. **b** Relative expression of FTO in bladder urothelial carcinoma cell lines (5637, T24, TCCSUP) and the normal bladder epithelial cell line (SV-HUC-1)

We analyzed the expression of FTO mRNA between the bladder urothelial carcinoma tissues and the normal controls from 30 patients following radical cystectomy in the Second Hospital of Dalian Medical University. FTO mRNA was significantly down-regulated in bladder urothelial carcinoma tissues (*P* = 0.0143, Fig. 1a). Western blot analysis showed an decrease in FTO expression in bladder cancer cell lines (5637, T24 and TCCSUP) compared with a normal bladder epithelial cell line (SV-HUC-1) (Fig. 1b).

### FTO mRNA expression in different stages (TNM) of bladder urothelial carcinoma samples

**Fig. 2:**
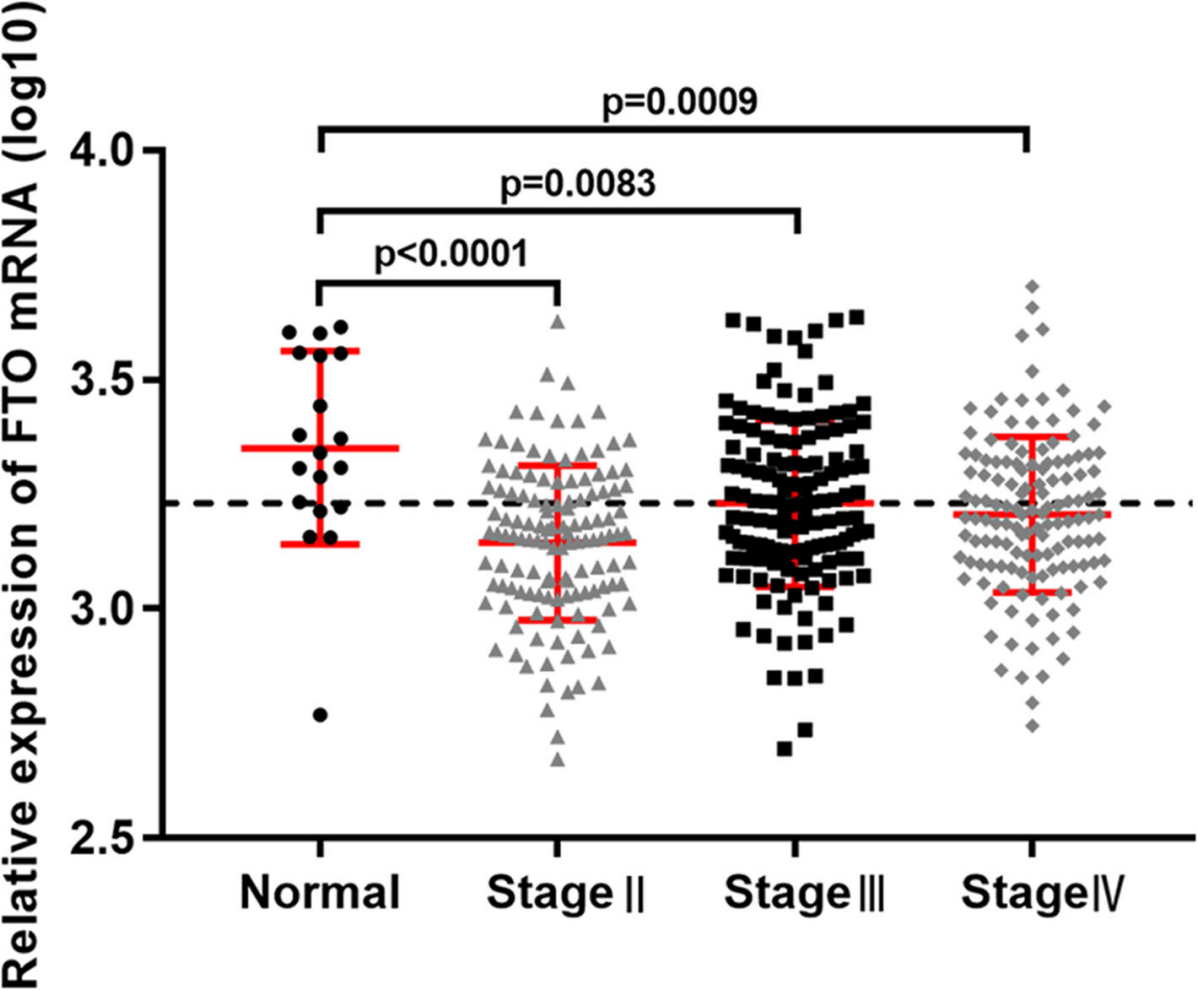
Expression of FTO mRNA in bladder urothelial carcinoma tissues compared with the normal controls from the data of TCGA

We analyzed the RNA-Seq data for FTO in different stages (TNM) of bladder urothelial carcinoma samples from TCGA (412 cancerous and 19 normal samples). Among the 412 cancerous samples, there were 2 without stage described, 4 of stageI, 129 of stageII, 142 of stageIII and 135 of stageIV. For the limitation of sample size, stageI was excluded for analysis of FTO mRNA expression between bladder urothelial carcinoma and the normal controls. The expression level of FTO mRNA was significantly down-regulated in stageII-IV of bladder urothelial carcinoma tissues compared with the normal controls (*p* < 0.0001 for stageII, *p* = 0.0083 for stageIII and *p* = 0.0009 for stageIV, Fig. 2).

### Downregulation of FTO promotes the proliferation and migration of bladder urothelial carcinoma cells

**Fig. 3:**
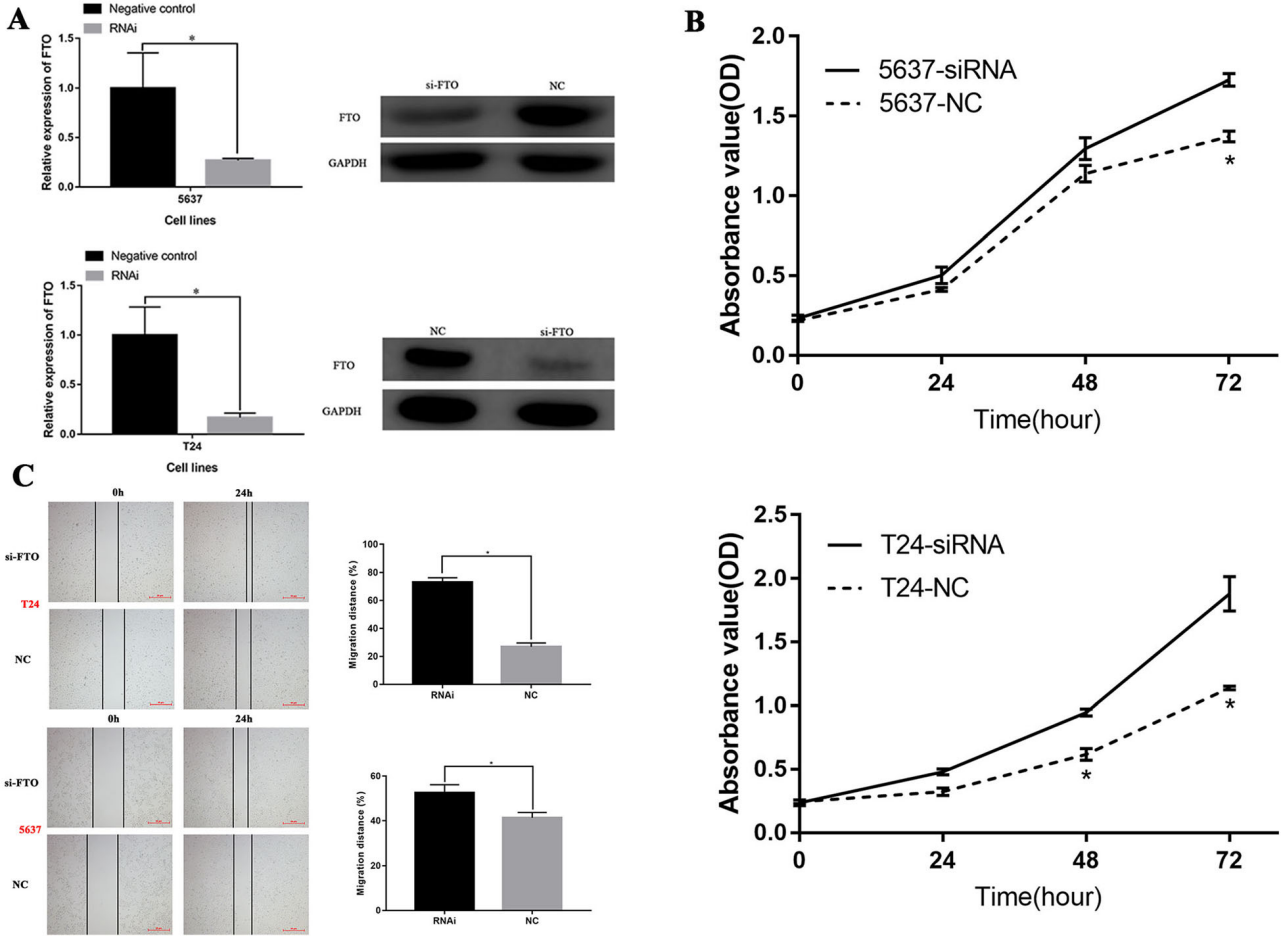
Downregulation of FTO promotes the proliferation and migration of bladder cancer cells**. a** Reverse transcription quantitative polymerase chain reaction assays and western bloting were used to determine the efficiency of RNAi transfection in 5637 and T24 cells. **b** The proliferation ability of 5637 and T24 cells after downregulation of FTO. **c** The migration ability of 5637 and T24 cells after downregulation of FTO. (*P < 0.05.). Abbreviation: OD, optical density; NC, negative control; RNAi, RNA interference

5637 and T24 cells were transfected with an siRNA targeting FTO to explore the role of FTO on cell proliferation and migration. At 16 h after transfection, FTO was strikingly downregulated in the RNAi group compared with the control group (*P* < 0.05; Fig. 3a). The CCK-8 results showed that si-FTO markedly promoted the proliferation of 5637 and T24 cells (P < 0.05; Fig. 3b). Wound-healing assays revealed that si-FTO increased the cell migration capacity (Fig. 3c). Overall, these data suggest that knockdown of FTO could promote the proliferation and migration of 5637 and T24 cells.

### The inhibitor of FTO (MA2) protects against cisplatin-induced cytotoxicity in bladder cancer cells

**Fig. 4:**
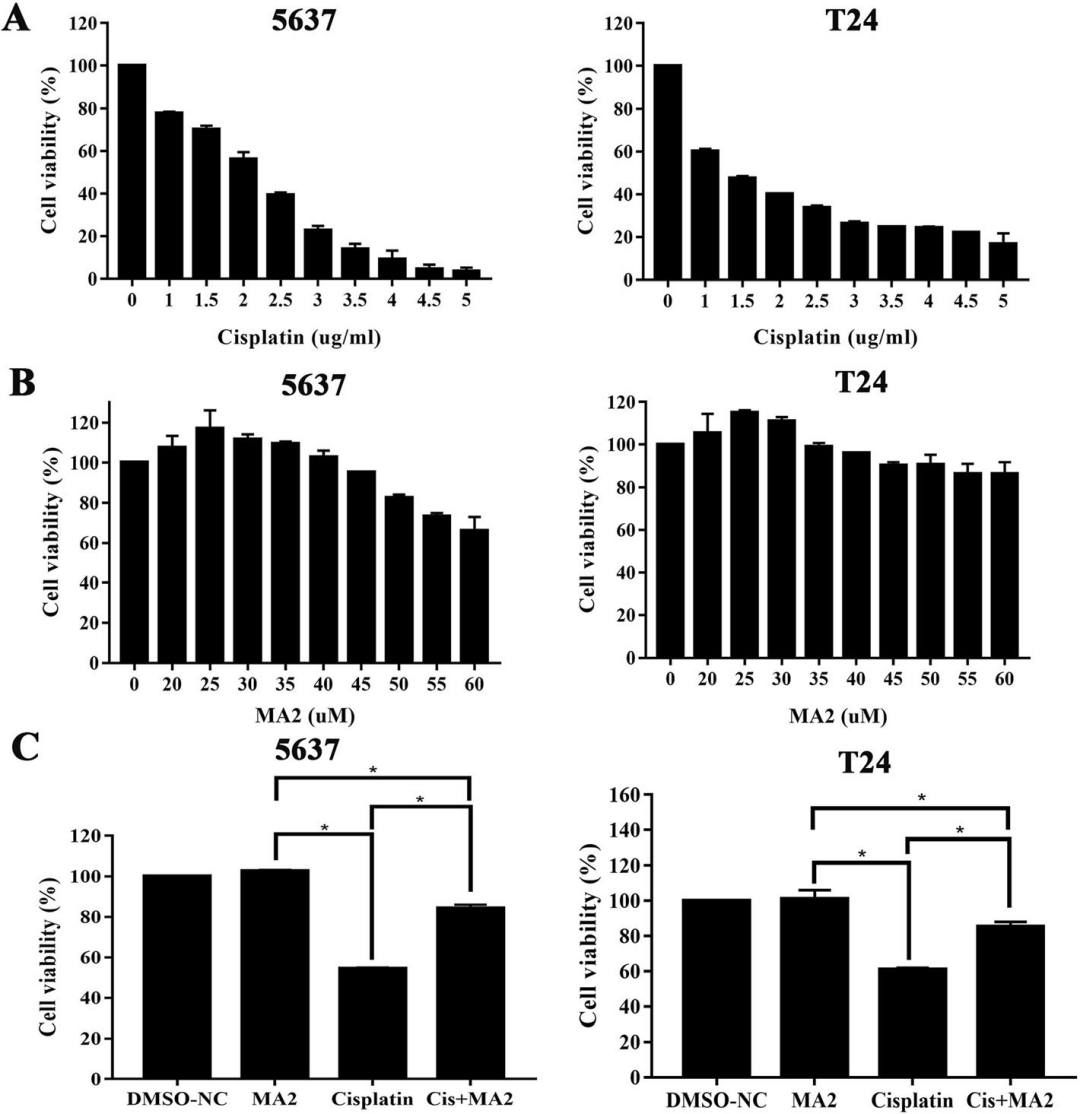
MA2 protects against cisplatin-induced cytotoxicity in bladder cancer cells**. a** 5637 and T24 cells were incubated with different concentrations of cisplatin for 48 h, and cell viability was measured with the CCK-8 kit. **b** 5637 and T24 cells were incubated with different concentrations of MA2 for 48 h, the cell viability was measured with the CCK-8 kit. **c** 5637 and T24 cells were incubated with selected concentration of cisplatin in the presence or absence of MA2 for 48 h, and the cell viability was measured with the CCK-8 kit. (*P < 0.05)

5637 and T24 cells were treated with different concentrations (0, 1, 1.5, 2, 2.5, 3, 3.5, 4, 4.5, and 5 μg/ml) for 48 h to determine the half maximal inhibitory concentration (IC50) of cisplatin for inducing bladder cancer cell death. The CCK-8 values for each cisplatin concentration were shown in Table 1 and Fig. 4a. 2 μg/ml and 1.5 μg/ml of cisplatin treatment were chosen as the IC50 of 5637 and T24 cells respectively. 5637 and T24 cells were treated with different MA2 concentrations (0, 20, 25, 30, 35, 40, 45, 50, 55, and 90 μM) for 48 h to determine the optimum concentration of MA2 treatment. The results of CCK-8 assay were shown in Table 2 and Fig. 4b. We choosed 50 μM and 35 μM MA2 for 5637 and T24 cells, which were the highest dose showing no significant cell death, for the following experiments, respectively. We divided the 5637 and T24 cells into four groups to explore the role of MA2 in cisplatin-induced injury subsequently, including the DMSO group, MA2 group, cisplatin group, and the cisplatin + MA2 group. Results showed that, compared with the control group, proportions of viable cells in the cisplatin group (54.49 ± 0.62% and 60.83 ± 1.63% for 5637 and T24, respectively) and the cisplatin+MA2 group (84.22 ± 2.53% and 85.45 ± 3.64 for 5637 and T24, respectively) decreased significantly (Fig. 4c). However, cell viability of 5637 and T24 could be rescued by co-culture with MA2 after cisplatin injury.

**Table 1:** The CCK-8 values for each cisplatin concentration on 5637 and T24 cells

Cisplatin concentration (μg/ml)	Cell viability (Mean + SD)	
	5637	T24
0	100%	100%
1	77.74 ± 1.1%	60.26 ± 1.54%
1.5	70.27 ± 2.33%	47.41 ± 1.63%
2	56.21 ± 4.66%	40.06 ± 0.45%
2.5	39.28 ± 1.85%	33.61 ± 1.63%
3	22.74 ± 3.02%	26.26 ± 1.54%
3.5	13.97 ± 3.5%	24.79 ± 0.18%
4	9.12 ± 5.83%	24.22 ± 0.99%
4.5	4.51 ± 3.02%	22.11 ± 0.18%
5	3.54 ± 2.47%	16.74 ± 7.05%

*Abbreviations***:***SD* standard deviation

**Table 2:** The CCK-8 values for each MA2 concentration on 5637 and T24 cells

MA2 concentration (μM)	Cell viability (Mean + SD)	
	5637	T24
0	100%	100%
20	107.37 ± 8.42%	105.36 ± 12.87%
25	116.9 ± 13.06%	114.91 ± 1.73%
30	111.5 ± 3.71%	111.04 ± 2.65%
35	109.49 ± 1.57%	98.9 ± 2.65%
40	102.67 ± 4.78%	95.93 ± 0.27%
45	95.26 ± 1.5%	90.25 ± 2.10%
50	82.57 ± 2.21%	90.64 ± 6.48%
55	73.26 ± 2.28%	86.38 ± 6.48%
60	65.99 ± 9.85%	86.38 ± 7.58%

*Abbreviations***:***SD* standard deviation

## Discussion

Bladder cancer shows a high frequency of relapse and poor prognosis once progress to muscle-invasive type. Patients with MIBC have a poor prognosis due to its aggressive nature and resistance to chemotherapy. Despite receiving curative surgery, approximately half of patients with MIBC develop metastatic disease within 2 years [15]. The standard drugs used in perioperative chemotherapy for MIBC and metastatic disease are cisplatin-based. Results of a randomized clinical trial involving patients after cystectomy showed that there were no statistically significant difference in OS between patients receiving cisplatin-based chemotherapy and patients for observation [16]. On the other hand, the objective response rate of chemotherapy treatment is only 40–60%, with median OS slightly > 1 year owing to chemoresistance [17, 18]. It has been demonstrated that multiple mechanisms are involved in chemoresistance and thus resulting in non-response of patients receiving chemotherapy [19]. For example, alterations in some genes (BCL2) could contribute to resistance to cisplatin in patients with muscle-invasive bladder cancer [20]. In the present study, we find that knockdown of FTO significantly promotes proliferation and migration of 5637 and T24 cells. Consistently, the cisplatin-induced cytotoxicity of bladder cancer cell could be rescued by co-treatment with MA2 [21], which was a highly selective inhibitor of FTO. Thus finding a way to regulate the expression of FTO, which could improve the sensitivity of cisplatin-based chemotherapy, may prolong the survival of patients.

As far as we know, we are the first one to report the FTO expression and its biological role in bladder urothelial carcinoma. Previous studies demonstrate a strong association between up-regulation of FTO and various cancers, including breast, prostate and kidney [22, 23]. It is possible that increased expression of FTO may contribute to the higher risk of individuals with overweight or obesity in developing cancers [24, 25]. Recent studies show that obesity-linked FTO mutations do not affect the FTO protein though they are the most common genetic contributor to obesity [26, 27]. Human obesity is linked to mutations within the FTO gene, which controls the expression of neighboring genes [28]. Li et al. showed that FTO could decrease the m6A level and increase mRNA stability of ubiquitin-specifific protease (USP7) by functioning as a demethylase in lung cancer cells [29]. Similarly, anonther paper reported that, in hepatocellular carcinoma, FTO could trigger the demethylation of PKM2 mRNA and accelerate the translated production [30]. In the present study, we surprisingly found that the FTO expression was down-regulated in bladder urothelial carcinoma. There are also findings that demonstrated the downregulation of FTO playing an oncogenic role in intrahepatic cholangiocarcinoma and clear cell renal cell carcinoma, respectively [31, 32]. TEAD2 mRNA stability was influenced by FTO in intrahepatic cholangiocarcinoma cells [31]. In clear cell renal cell carcinoma, FTO could inhibit tumour growth by reducing m6A levels in PGC-1α mRNA transcripts [32]. In the present study, we found that the cisplatin-induced cytotoxicity of bladder cancer cells could be rescued by co-treatment with MA2, which was a highly selective inhibitor of FTO. Yang’s study also demonstrated that knockdown of FTO sensitized melanoma cells to interferon gamma and anti-PD-1 treatments depending on adaptive immunity [33].

Although the m6A profile could be mediated by the m6A eraser FTO, it can also be determined by m6A writer methyltransferase-Like 3 (METTL3) [34]. This reversible RNA modification is particularly exciting since it raises the possibility that RNA modifications would be formed and removed in a dynamic manner. Li et al. showed that FTO is a novel oncogene that promotes AML in their study [13]. However, another study proved that m6A writers have an oncogene-like effect in AML [35]. Results showed that FTO functioning as an oncogene are inconsistent with the CRISPR screens in AML lines. It seems that elevated m6A has an oncogenic effect. The findings in our study indicates that down-regulation of FTO plays an oncogenic role, which may indicate a high m6A level in bladder urothelial carcinoma. The presumably RNA demethylase activity of FTO may has important roles in physiological processes in bladder urothelial carcinoma. Thus, we suppose that FTO may play its role in bladder urothelial carcinoma as an RNA demethylase in the reversible RNA modification.

In our study, in vitro cell lines and clinical samples were used. Although we have conducted a wide range of analyses using data from TCGA to improve our understanding of the relationship between FTO and bladder urothelial carcinoma, some limitations were hard to avoid. First, to fully understand the relationship between FTO and the development of bladder urothelial carcinoma, the effect of overexpression of FTO on 5637 and T24 cells are needed. Second, we did not investigate effects on m6A demethylation following siRNA or pharmacological inhibition of FTO, which need our further exploration.

## Conclusions

In conclusion, these findings suggest that down-regulation of FTO plays an oncogenic role in bladder cancer. The further exploration of regulation of FTO expression may provide us a potential therapeutic target for the treatment of bladder cancer.

## Data Availability

The datasets used and/or analyzed during the current study available from the corresponding author on reasonable request.

## Abbreviations

FTO: Fat mass and obesity associated protein
PCR: Real-time polymerase chain reaction;
TCGA: the Caner Genome Atlas
MA2: meclofenamic acid
BC: bladder cancer
AML: acute myeloid leukemia
BSA: bovine serum albumin
CCK8: Cell Counting Kit-8
DMSO: dimethyl sulfoxide
MIBC: muscle invasive bladder cancer
SV-HUC-1: SV-40 immortalized human uroepithelial cell line
T24 and 5637: bladder cancer cell lines
OS: overall survival.
